# Atorvastatin Enhances the Efficacy of Immune Checkpoint Therapy and Suppresses the Cellular and Extracellular Vesicle PD-L1

**DOI:** 10.3390/pharmaceutics14081660

**Published:** 2022-08-09

**Authors:** Eun-Ji Choe, Chan-Hyeong Lee, Ju-Hyun Bae, Ju-Mi Park, Seong-Sik Park, Moon-Chang Baek

**Affiliations:** Department of Molecular Medicine, CMRI, Exosome Convergence Research Center (ECRC), School of Medicine, Kyungpook National University, Daegu 41944, Korea

**Keywords:** statin, atorvastatin, extracellular vesicles, EV PD-L1, immunotherapy

## Abstract

According to clinical studies, statins improve the efficacy of programmed death-1/programmed death-ligand 1 (PD-1/PD-L1) blockade therapy for breast cancer; however, the underlying mechanisms are unclear. Herein, we showed that atorvastatin (ATO) decreased the content of PD-L1 in extracellular vesicles (EVs) by reducing cellular PD-L1 expression and inhibiting EV secretion in breast cancer cells, thereby enhancing the efficacy of anti-PD-L1 therapy. ATO reduced EV secretion by regulating the Rab proteins involved in EV biogenesis and secretion. ATO-mediated inhibition of the Ras-activated MAPK signaling pathway downregulated PD-L1 expression. In addition, ATO strongly promoted antitumor efficacy by inducing T cell-mediated tumor destruction when combined with an anti-PD-L1 antibody. Moreover, suppression of EV PD-L1 by ATO improved the reactivity of anti-PD-L1 therapy by enhancing T-cell activity in draining lymph nodes of EMT6-bearing immunocompetent mice. Therefore, ATO is a potential therapeutic drug that improves antitumor immunity by inhibiting EV PD-L1, particularly in response to immune escape during cancer.

## 1. Introduction

Immunotherapy with current programmed death-1/programmed death-ligand 1 (PD-1/PD-L1) inhibitors has been approved for clinical application because of its promising advantages in improving the prognosis of cancer patients. However, the responses of many patients to immunotherapy remain unsatisfactory [1]. PD-1 bound to PD-L1 can inhibit T-cell activation. Tumor cells overexpress PD-L1 and bind the PD-1 of T cells, thereby causing immune escape. The use of anti-PD-1/PD-L1 antibodies prevents their binding to some extent and partially restores T-cell activity. PD-L1 can be expressed on the surface of tumor cells; however, it also exists in extracellular forms, such as extracellular vesicles (EVs) including exosomes. EV PD-L1 has the same structure as that of PD-L1 on the tumor cell surface; it can also bind to PD-1 in T cells. EV PD-L1 that binds to PD-1 on T cells inhibits the activation and proliferation of CD8^+^ T cells, and can cause immune evasion [2,3].

Abnormal synthesis of mevalonate and cholesterol is closely related to tumor development, growth, and progression [4,5]. Cholesterol is implicated in the clinical progression of breast cancer. Previous studies have identified the cholesterol metabolite 27-hydroxycholesterol (27HC) as a major mediator of the effects of cholesterol on breast tumor growth and progression [6]. 27HC acts as an estrogen receptor (ER) modulator to promote the growth of ERα^+^ tumors and as a liver X receptor ligand in myeloid immune cells to establish an immunosuppressive program. 27HC promotes EV secretion [6]. The secretion and contents of EVs can be regulated by a cholesterol metabolite, which may have detrimental effects on disease progression.

Statins are cholesterol inhibitors that selectively inhibit HMG-CoA reductase (3-hydroxy-3-methyl-glutaryl coenzyme A reductase, HMGCR), a rate-limiting enzyme in the mevalonate biosynthesis pathway; they are widely used in treating hyperlipidemia and cardiovascular diseases [7]. Statins have beneficial effects, such as improving overall survival and reducing the risk of cancer-related deaths; however, their potential effects on cancer remain uncertain [8,9,10]. In addition, elevated anticancer effects of statins in chemotherapy and targeted therapy are observed in patients with various cancers [11,12]. Statins can be used in combination with immune checkpoint blockade therapy in cancer. In particular, statins exhibit significantly improved clinical results in patients with progressive non-small cell lung cancer (NSCLC) treated with nivolumab, a PD-1-targeted monoclonal antibody [13]. However, statin therapy is not an independent favorable prognostic factor, although it is correlated with a favorable outcome [14].

Recently, statins have been reported to reduce PD-L1 by inhibiting AKT and β-catenin signaling in NSCLC [15]. Statins are also reported to block the secretion of EVs induced by upregulating cholesterol metabolism in acute myeloid leukemia and reduce exosome secretion by inhibiting the mevalonate pathway in breast cancer [16,17]. However, the function of statins in immune checkpoint regulation or EV PD-L1-mediated immune regulation in cancer is largely unexplored. Based on the above factors, we hypothesize that statins could affect EV PD-L1 expression, which is involved in immune evasion in cancers.

In this study, we found that atorvastatin (ATO) combined with anti-PD-1/PD-L1 antibodies can inhibit EV PD-L1, promote cytotoxic T-cell activity, and enhance antitumor immune response, thereby causing antitumor effects.

## 2. Materials and Methods

### 2.1. Cell Lines and Cell Culture

All the human and mouse cancer cells were purchased from the American Type Culture Collection. The cells were grown at 37 °C under a humidified atmosphere with 5% CO_2_ and 95% air. MDA-MB-231 and LLC1 cells were maintained in Dulbecco’s Modified Eagle Medium supplemented with 10% fetal bovine serum (FBS) and 1% antibiotics. EMT6, 4T1, and CT26 cells were maintained in RPMI supplemented with 10% FBS and 1% antibiotics. The cell lines were ensured to be free of mycoplasma contamination before any experiments were performed. The cells were changed every 30 days to maintain their condition.

### 2.2. Transmission Electron Microscopic Imaging

The EVs of MDA-MB-231 cells isolated by ultracentrifugation were deposited on pure carbon-coated EM grids. The cells were fixed with 2% paraformaldehyde for 5 min and washed thrice with phosphate-buffered saline (PBS). The cells were visualized at 40,000× magnification [1] and 100 kV using a Hitachi HT-7700 transmission electron microscope (Tokyo, Japan).

### 2.3. Cell Cytotoxicity Assay

MDA-MB-231 cells (50,000 cells/well) and EMT6 cells (10,000 cells/well) were seeded onto 24-well cell culture plates and incubated for 24 h. The medium was replaced with a serum-free medium with different concentrations of statins for another 48 h. Next, 0.5 mg/mL of MTT [3-(4,5-dimethylthiazol-2-yl)-2,5-diphenyltetrazolium bromide] solution was added, and the MTT solution and medium were removed. Isopropanol was added and formazan crystals were dissolved by gentle shaking at room temperature. The mixture was transferred to a 96-well plate and the absorbance was measured at 595 nm using a microplate reader.

### 2.4. Isolation of EVs

EVs were isolated following the method previously described [18,19]. MDA-MB-231 and EMT6 cells were seeded onto a 15-cm plate (5 × 10^6^ cells/plate). The supernatants were centrifuged at 300× *g* for 3 min, 2500× *g* for 15 min, and 10,000× *g* for 30 min. Then, the supernatants were filtrated using a 0.2-mm syringe filter and centrifuged at 120,000× *g* for 90 min. The pellets containing EVs were resuspended in 1 mL PBS and examined using nanoparticle tracking analysis (NTA). The EV pellets were resuspended in a PBS or 1× radioimmunoprecipitation assay (RIPA) buffer for further experiments.

Mouse plasma was centrifuged at 2500× *g* for 15 min and 10,000× *g* for 30 min. Then, the supernatants were centrifuged at 120,000× *g* for 90 min. The pellets containing EVs were resuspended in a 1× RIPA buffer (Cell Signaling Technology, Danvers, MA, USA). Protein contents in the EVs were quantified using a Pierce BCA protein assay kit (Thermo Scientific, Waltham, MA, USA). The purity range was approximately 2.5–3.8 × 10^8^ EVs/μg.

### 2.5. NTA

Cell culture supernatants containing EVs were analyzed using a NanoSight LM10 device (NanoSight, Malvern, UK). A monochromatic laser beam at 405 nm was used to analyze the nanoparticles; a video of 30-s duration was recorded at a rate of 30 frames/s and a camera level of 9. EV movement was analyzed using NTA software (version 2.0; NanoSight). Approximately 30–100 particles were analyzed for each field of view. 

### 2.6. Western Blot Analysis

Proteins derived from cells and EVs were resolved using sodium dodecyl sulfate polyacrylamide gel electrophoresis (SDS–PAGE), transferred onto nitrocellulose membranes, bound with primary antibodies, and incubated with horseradish peroxidase (HRP)-conjugated secondary antibodies. Protein bands were visualized using enhanced chemiluminescence detection reagents (34580, Thermo Scientific, Waltham, MA, USA). Primary antibodies against the following proteins were used: Alix (ab56932, Abcam, Cambridge, UK), CD63 (ab68418, Abcam), TSG101 (ab56932, Abcam), Synthenin-1 (ab133267, Abcam), β-actin (112620, Cell Signaling Technology, Danvers, MA, USA), PD-L1 (13684, Cell Signaling Technology), Rab27a (ab55667, Abcam), Rab11 (ab18211, Abcam), Rab7 (ab50533, Abcam), pan Ras (sc-166691, Santa Cruz, Santa Cruz, CA, USA), Rap1A (sc-398755, Santa Cruz), p-B-Raf (2696S, Cell Signaling Technology), B-Raf (9433S, Cell Signaling Technology), p-MEK (9154S, Cell Signaling Technology), MEK (9126S, Cell Signaling Technology), p-ERK1/2 (4370S, Cell Signaling Technology), ERK1/2 (4695S, Cell Signaling Technology), p-AKT (4060S, Cell Signaling Technology), and AKT (9272S, Cell Signaling Technology).

### 2.7. Flow Cytometry Analysis

MDA-MB-231 and EMT6 cells were harvested by centrifugation at 300× *g* for 3 min. For cell surface staining, cell suspensions were washed in PBS, stained with an anti-PD-L1 antibody for 15 min on ice, and washed with PBS. The mean fluorescence intensity of the cell surface PD-L1 was measured using a CytoFLEX instrument (Beckman Coulter, Brea, CA, USA).

### 2.8. CD8^+^ T Cell-Mediated Cancer Cell Killing Assay

MDA-MB-231-luciferase cells (5000 cells/well) and 4T1-luciferase cells (5000 cells/well) were plated in a 96-well plate. Human CD8^+^ T cells were isolated from human peripheral blood mononuclear cells (PBMCs). Murine CD8^+^ T cells were isolated from the spleen of healthy BALB/c mice. Cancer cells were then co-cultured with activated human CD8^+^ T and murine CD8^+^ T cells. Next, the cells were treated with ATO and αPD-L1 for 48 h at an effector-to-target ratio of 1:5. The cells were washed with PBS and 100 µL of 2 mg/mL luciferin was added to each well. Luciferase activity was immediately measured using an Alpha microplate reader (PerkinElmer, Waltham, MA, USA).

### 2.9. Animal Experiments

All the animal experiments were performed in accordance with the protocols approved by the Kyungpook National University (KNU) Institutional Animal Care and Use Committee (IACUC). EMT6 cells (2 × 10^5^ cells in 50 μL Matrigel with 50 μL PBS) were injected into the left fat pad of five-week-old female BALB/C nude mice. ATO (0.5, 1, and 10 mg·kg^−1^·day^−1^) was intragastrically administered for 21 days. EMT6 cells (2 × 10^5^ cells in 50 mL Matrigel with 50 mL PBS) were injected into the left fat pad of five-week-old female BALB/C wild-type mice. An anti–PD-L1 antibody (200 μg/mouse) was administered intraperitoneally, and ATO (1 and 10 mg·kg^−1^·day^−1^) was administered intragastrically. Tumor volume was measured every three days using a digital caliper and calculated using the formula: (width)^2^ × length/2.

Mice were euthanized when their tumor volumes reached 1500 mm^3^. Complete regression was defined as the tumors below 50 mm^3^ and continuing to regress until the end of the study. A rechallenge experiment was performed as described previously [20]. 

### 2.10. Immune Phenotyping and Flow Cytometry Analysis

Flow cytometry immune phenotyping was performed as previously described [18]. Mice were sacrificed on day 21 and tumor-draining lymph nodes (TDLNs) were harvested. The dead cells isolated from TDLNs were stained using a fixable aqua dead cell stain kit (Invitrogen, Waltham, MA, USA) for 30 min on ice. The samples were first stained for surface markers for lymphoid and immune populations followed by intracellular staining. For FoxP3 and intracellular staining, a Foxp3/Transcription factor staining buffer set (eBioscience, San Diego, CA, USA) was used according to the manufacturer’s protocol. The samples were blocked with Fc block (BD Pharmingen, San Diego, CA, USA) for 10 min on ice before staining with antibodies. Fluorescein isothiocyanate (FITC) anti-mouse CD3, efluor780 anti-mouse CD45, peridinin chlorophyll protein-Cyanine 5.5 anti-mouse CD4, efluor450 anti-mouse CD8, APC anti-mouse GzmB, APC anti-mouse IFNγ, and PE anti-mouse FoxP3 were used. Immune phenotyping was performed using a CytoFLEX system (Beckman Coulter). Single-dye staining was performed for compensation controls, and nonspecific binding was evaluated with isotype controls.

### 2.11. Statistical Analysis

An unpaired two-tailed Student’s *t*-test was used to compare the two sets of data. Data in the graph represent mean ± standard deviation. Statistical significance was set at *p* < 0.05; *, **, ***, and **** correspond to *p*-values < 0.05, <0.01, <0.001, and <0.0001, respectively. NS indicates no significant difference. Data were analyzed using the PRISM 6 software (GraphPad Software, Inc., San Diego, CA, USA).

## 3. Results

### 3.1. ATO Suppresses EV PD-L1 by Inhibiting EV Secretion and PD-L1 Expression

**Figure 1:**
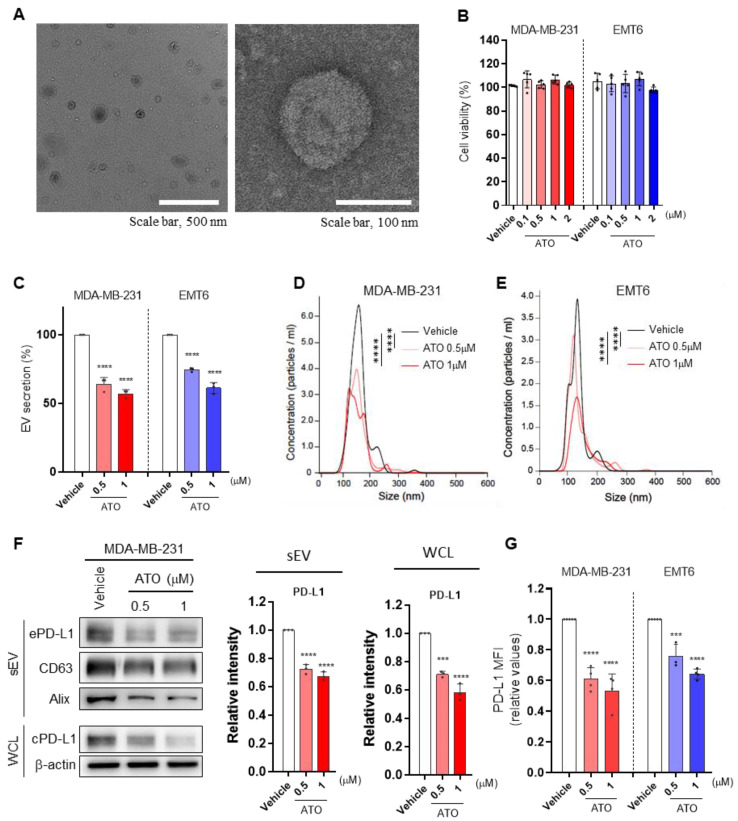
Atorvastatin (ATO) suppresses the secretion of EV PD-L1 by inhibiting EV secretion and PD-L1 expression. (**A**) Transmission electron microscopy images of MDA-MB-231-derived EVs. The images at the left and right panels show EVs with low (scale bar, 500 nm) and high magnification (scale bar, 100 nm), respectively. (**B**) Measurement of cancer cell viability by MTT assay. MDA-MB-231 and EMT6 cells were treated with different concentrations of ATO (*n* = 5). (**C**) Measurement of secreted EVs by MDA-MB-231 and EMT6 cells using nanoparticle tracking analysis (NTA). Quantification of secreted EVs in the presence of different ATO concentrations (*n* = 3). Size distribution of secreted EVs from (**D**) MDA-MB-231 and (**E**) EMT6 cells with or without ATO treatment assessed using NTA. (**F**) Immunoblot of various proteins in EVs and whole-cell lysates from MDA-MB-231 cells with or without ATO treatment (*n* = 3). (**G**) Fold change of mean fluorescence intensity (MFI) of PD-L1 levels. The results are presented as means ± S.D. of three independent experiments. *** *p*  <  0.001, and **** *p*  <  0.0001.

To determine whether ATO affects tumor-derived EV PD-L1, we treated breast cancer cell lines, including MDA-MB-231 and EMT6 cells, with different ATO concentrations and measured EV secretion. We confirmed the morphology of EVs secreted from MDA-MB-231 cells (Figure 1A). We determined a drug concentration that did not induce cytotoxicity. The MTT assay showed no cell death at different ATO concentrations used here (Figure 1B). ATO significantly inhibited EV secretion in a dose-dependent manner without affecting the particle size (Figure 1C–E). Next, we investigated the effects of ATO on EV secretion-related protein levels. As EV secretion decreased, the levels of EV marker proteins, such as CD63 and Alix, decreased during ATO treatment. In addition, PD-L1 levels significantly decreased in lysates of ATO-treated cells, and cell surface PD-L1 expression was also decreased (Figure 1F,G). Therefore, ATO decreased EV PD-L1 expression by preventing EV secretion and reducing PD-L1 expression in tumor cells.

### 3.2. ATO Suppresses EV PD-L1 by Regulating EV Biogenesis and the Mevalonate Pathway

**Figure 2:**
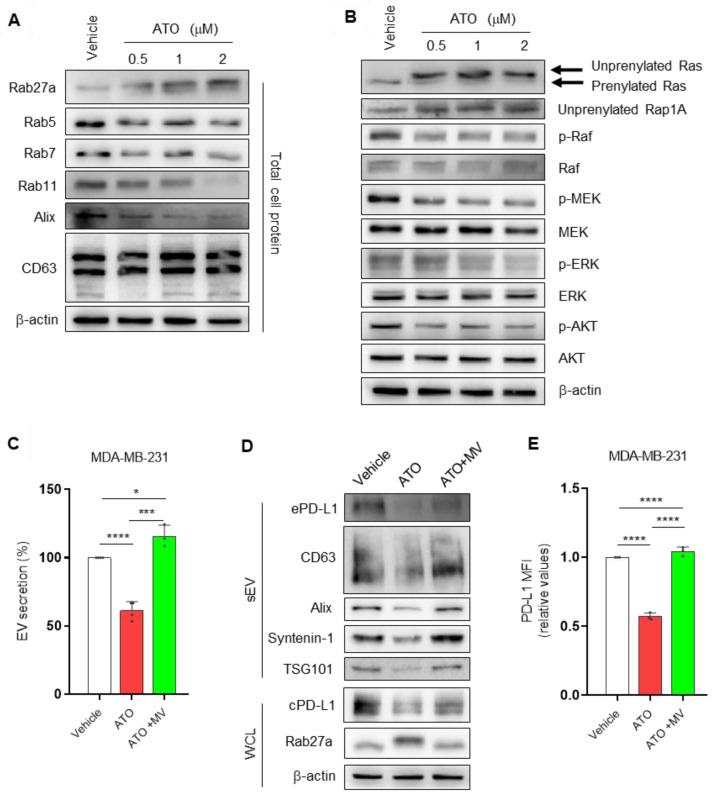
ATO suppresses EV PD-L1 by regulating EV biogenesis and the mevalonate pathway. (**A**) Immunoblot for the indicated Rab family proteins (Rab27a, Rab5, Rab7) and MVB (multivesicular body) marker (Alix, CD63) in MDA-MB-231 cells. β-actin was used as a loading control for whole-cell lysates. (**B**) Changes in Ras, Rap1A, Raf, MEK, ERK, and AKT expression in MDA-MB-231 cells. (**C**) The inhibitory effect of ATO on EV secretion was reversed by the addition of mevalonic acid (MV) to MDA-MB-231 cells. (**D**) The inhibitory effects of ATO on the expression of EV and other proteins in MDA-MB-231 cells were reversed by the addition of MV. (**E**) The inhibitory effect of ATO on surface PD-L1 levels was reversed by the addition of MV to MDA-MB-231 cells. The results are presented as means ± S.D. of three independent experiments. * *p* < 0.05, *** *p* < 0.001, and **** *p* < 0.0001.

To further explore the potential mechanism of EV PD-L1 inhibition by ATO, we examined whether ATO influences EV biogenesis. We investigated the expression of Rab and EV secretion-related proteins by western blotting. When MDA-MB-231 cells were treated with ATO, which inhibits prenylation by inhibiting HMGCR, the location of Rab27a (an EV secretion-related marker) shifted in blots, and the expression of Rab5, Rab7 (early and late endosome markers), and Rab11 (recycling endosome marker) decreased [21]. Interestingly, we observed a single band for the untreated cells; however, an additional mobility-shifted isoform of Rab27a was noticed in ATO-treated cells. This isoform may represent un-prenylated Rab27a because the electrophoretic mobility of unmodified Rab27a proteins is slower than that of the prenylated forms in SDS–PAGE [22]. Moreover, the expression of Alix (multivesicular body formation protein), which is involved in EV release, decreased by ATO treatment. No significant difference in the expression of CD63 was noticed (Figure 2A). Therefore, ATO inhibited EV release by inhibiting EV biogenesis and secretion.

Atorvastatin inhibits Ras activation and its downstream signaling in glioblastoma cells [23]. PD-L1 expression is mainly regulated via MAPK (Ras/Raf/MEK/ERK) and PI3K/Akt pathways and can be controlled by many intracellular and extracellular signals [24,25]. Therefore, we investigated whether the inhibitory effect of ATO on the PD-L1 expression of breast cancer cells was associated with these signaling pathways. We observed the unprenylated form of Ras with reduced electrophoretic mobility. Moreover, increased unprenylated forms of Rap1A were detected in MDA-MB231 cells [26]. Consistent with the inhibition of Ras prenylation, ATO decreased the phosphorylation of downstream effectors of Ras signaling, such as Raf, MEK, ERK, and AKT (Figure 2B). Collectively, ATO significantly reduced PD-L1 expression and the phosphorylation of Ras, Raf, MEK, ERK, and AKT at the protein level.

Statins, as mevalonate pathway inhibitors, reportedly reduce cancer-specific mortality in patients with breast cancer [27]; however, the effects are controversial. HMGCR, a rate-limiting enzyme of the mevalonate pathway, is associated with the anticancer effects of statins [28]. Therefore, we hypothesized that the effects of ATO on EV PD-L1 levels were mediated by HMG-CoA reductase inhibition. We confirmed that the effects of ATO on EV secretion and cellular PD-L1 expression were inhibited by the addition of exogenous mevalonic acid (MV), the product of HMG-CoA reductase (Figure 2C,D). Additionally, the same phenomenon was observed for PD-L1 levels at the cell surface (Figure 2E). Moreover, prenylation of Rab27a, a protein involved in EV secretion, was restored with MV treatment, resulting in a band shift similar to that of the vehicle group (Figure 2D). Overall, these observations suggested that ATO-induced EV PD-L1 reduction is related to EV biogenesis and the mevalonate pathway. 

### 3.3. ATO Combined with Anti-PD-L1 Antibody Enhanced CD8^+^ T Cell-Mediated Cytotoxic Effects

**Figure 3:**
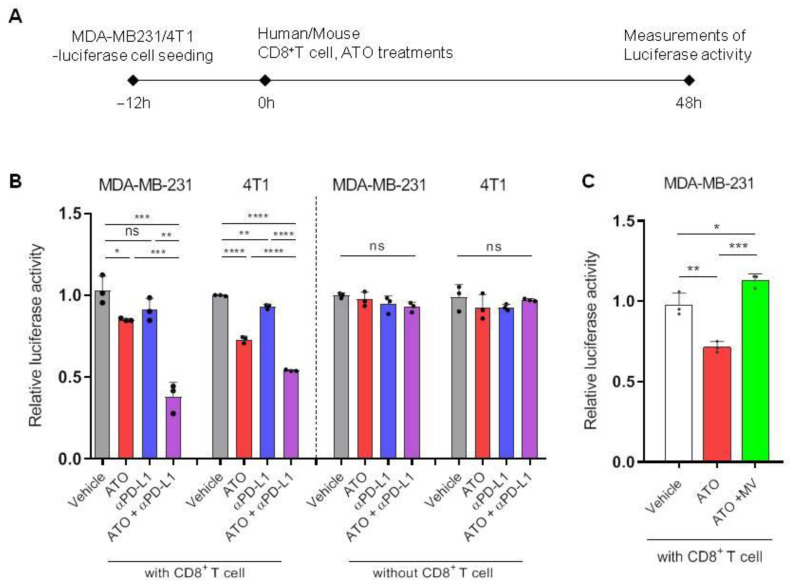
ATO combined with an anti-PD-L1 antibody enhances CD8^+^ T cell-mediated cytotoxic effects. (**A**) The experimental designs of the co-culture system to test CD8^+^ T-cell activity. (**B**) PBMC and mouse CD8^+^ T cell-mediated cytotoxicity in MDA-MB-231- and 4T1-luciferase cells following the indicated treatments (*n* = 3) (left). Luciferase activity of the 4T1-luciferase cells without mouse CD8^+^ T cells (*n* = 3) (right). (**C**) Mouse CD8^+^ T cell-mediated cytotoxicity of ATO reversed by the addition of MV. The results are presented as mean ± S.D. of three independent experiments. * *p* < 0.05, ** *p* < 0.005, *** *p* < 0.001, and **** *p* < 0.0001, respectively; NS, not significant.

In a previous experiment, we observed that cellular PD-L1 level, EV secretion, and EV PD-L1 decreased during ATO treatment in breast cancer cells (Figure 1C–E). Accordingly, we tested whether CD8^+^ T cells activated by ATO have increased tumor-killing potential in a co-culture system. MDA-MB-231- and 4T1-luciferase cells were co-cultured with CD8^+^ T cells isolated from human PBMCs and mouse spleen, respectively. After activating the T cells with anti-CD3 and CD28, they were treated with ATO and an anti-PD-L1 antibody. Luciferase activity was measured after a specific period (Figure 3A). The cytotoxic effect of CD8^+^ T cells increased when treated with a combination of ATO and an anti-PD-L1 antibody rather than ATO alone. Moreover, the combined treatment of ATO and an anti-PD-L1 antibody showed cytotoxic effects by enhancing T-cell activity without cancer cell death through the direct cytotoxic effect of these two drugs (Figure 3B). We also investigated whether the activity of CD8^+^ T cells was modulated by MV. CD8^+^ T cell-mediated cytotoxic effects were reversed when MV was added to anti-CD3/CD28- and ATO-treated cultures (Figure 3C). These observations indicate the potential of ATO in activating CD8^+^ T cells via the mevalonate pathway, enhancing their antitumor efficacy.

### 3.4. ATO Treatment Improves the Therapeutic Effect of Anti-PD-L1 Antibody

**Figure 4:**
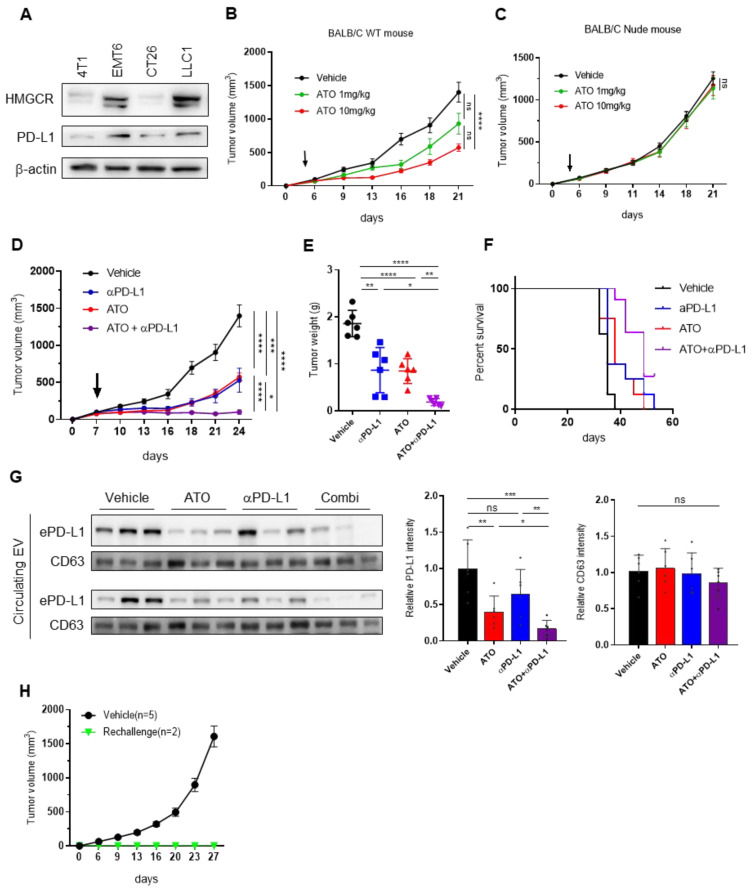
ATO treatment improves the efficacy of anti-PD-L1 immunotherapy. (**A**) Immunoblot for HMG-CoA reductase (HMGCR) and PD-L1 from mouse cancer cell lines. β-actin was used as a loading control for whole-cell lysates. EMT6 cells were subcutaneously inoculated into BALB/c (**B**) nude or (**C**) wild-type (WT) mice (*n* = 6). The arrows indicate the beginning of ATO administration (at tumor volume = 50–100 mm^3^). (**D**) Tumor mass of BALB/C WT mice implanted with EMT6 cells and treated with vehicle or ATO 10 mg/kg and anti-PD-L1 (*n* = 6). (**E**) Tumor weight of BALB/C WT mice implanted with EMT6 cells. (**F**) Survival analysis of BALB/C WT mice with different treatments (*n* = 6). (**G**) Immunoblots of PD-L1 of the circulating EVs and cellular PD-L1 in tumors treated with or without ATO. Quantitative analysis of relative protein expression (*n* = 6). (**H**) Changes in tumor volumes after tumor rechallenge in the EMT6 models. The results are presented as mean ± S.D. of three independent experiments. * *p* < 0.05, ** *p* < 0.005, *** *p* < 0.001, and **** *p* < 0.0001; NS, not significant.

We investigated whether ATO enhances antitumor effects by immune checkpoint blockade (ICB). To select mouse cancer cells before proceeding with the in vivo experiment, we studied the expression of PD-L1 and HMGCR, the target receptors of ATO, in several mouse cancer cell lines, including breast (4T1, EMT6), colorectal (CT26), and lung (LLC1) cancer cells. We found that both PD-L1 and HMGCR expression was highest in EMT6 cells (Figure 4A).

To investigate the effect of ATO on antitumor immunity, we compared the antitumor effects of ATO in immunocompetent BALB/c wild-type (WT) and immunodeficient nude mice using an EMT6 mouse model. We observed that tumor growth was significantly reduced in the ATO-administered group of WT mice; however, no significant change was observed in tumor growth in nude mice treated with 1 and 10 mg/kg ATO (Figure 4B,C). These results suggest that the antitumor effect of ATO depends on an intact T-cell response. Based on these observations, we hypothesized that ATO could enhance the antitumor effect of ICB therapy by inhibiting EV PD-L1. We investigated the effect of the combined treatment of ATO and an anti-PD-L1 antibody in the EMT6 syngeneic mouse model. The combination significantly improved tumor growth inhibition, as shown by the graphs of tumor volume and tumor weight (Figure 4D,E); in addition, it led to a higher survival rate than that of the vehicle or ATO, or anti-PD-L1 antibody treatment alone (Figure 4F). EV PD-L1 in mouse plasma decreased by ATO treatment alone or in combination with an anti-PD-L1 antibody. The combined treatment of ATO and an anti-PD-L1 antibody enhanced the therapeutic effect by suppressing EV PD-L1 in EMT6-bearing mice (Figure 4G). Additionally, to determine whether combination therapy with ATO and an anti-PD-L1 antibody induces a protective memory immune response, another set of tumors was rechallenged in mice whose tumors regressed after combination therapy in the EMT6 syngeneic tumor model. In the vehicle group, the tumor volume increased in a time-dependent manner, whereas in the rechallenged group, tumors no longer grew (Figure 4H). These results indicated that the combination of ATO and anti-PD-L1 induced immunological memory in the EMT6 model.

### 3.5. Combination Therapy with ATO and Anti-PD-L1 Antibody Increases Immune Response

**Figure 5:**
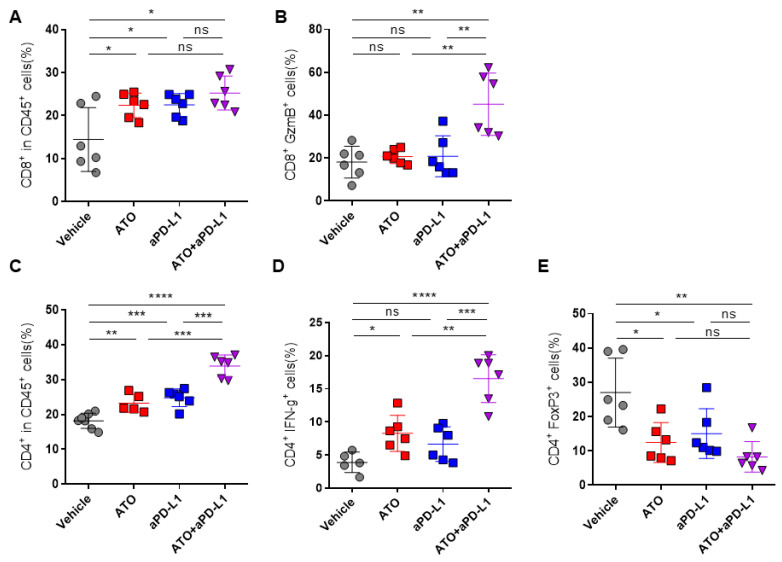
The combination therapy of ATO and an anti-PD-L1 antibody increases immune response. (**A**–**E**) Flow cytometry analysis of T lymphocytes in draining lymph nodes (DLNs) isolated from EMT6 bearing a mouse (*n* = 6). The proportions of CD8^+^ cells in (**A**) CD45^+^ and (**B**) GzmB^+^ cells in DLNs. The proportions of CD4^+^ cells in (**C**) CD45, (**D**) IFN-γ^+^, and (**E**) FoxP3^+^ cells in DLNs. The results are presented as mean ± S.D. of three independent experiments. * *p* < 0.05, ** *p* < 0.005, *** *p* < 0.001, and **** *p* < 0.0001; NS, not significant.

We explored the effect of ATO treatment on the function of CD8^+^ T cells in DLNs. The CD8^+^ T cell population and their cytotoxic activity significantly increased with the combination therapy of ATO and an anti-PD-L1 antibody (Figure 5A,B), and the population of CD4^+^ cells also increased; however, the proportion of regulatory T cells (Tregs) that suppress the effector T cells decreased (Figure 5C–E). Overall, ATO-mediated inhibition of tumor-derived EV PD-L1 enhanced CD8^+^ T cell-mediated antitumor immune response. This result suggests that ATO could be a potential therapeutic agent with anti-PD-L1 in clinical settings.

## 4. Discussion

In recent years, ICB therapy, which blocks immune checkpoints including PD-1/PD-L1, has shown promising applications in clinical settings. However, with patient responses < 20% [3], new therapeutic strategies are required to improve the efficacy of immune checkpoint inhibitors.

Cholesterol is an essential component of mammalian cell membranes. Highly proliferative cells, such as cancer cells, require more cholesterol to quickly synthesize membranes [29]. Statins may influence tumor proliferation and exhibit anticancer activity by reducing intracellular cholesterol biosynthesis. Although epidemiological evidence regarding the role of statins in cancer patients is ambiguous [30], multiple studies have shown that statins inhibit tumor development by targeting the L-mevalonate pathway [31,32,33]. Moreover, inhibition of the mevalonate pathway by statins in breast cancer cells decreases exosome secretion [17]. However, the mechanisms of the in vitro antitumor potential of ATO are unclear. EVs have emerged as one of the key components regulating the overall response to anti-PD-1/PD-L1 therapy [2]. EVs act as important mediators in cancer initiation, progression, metastasis, and immunity [34]. In addition, in a recent clinical trial, combinatorial therapy using nivolumab and statin improved NSCLC survival rates; however, information regarding the functions of ATO in immune checkpoint regulation in cancer or EV PD-L1-mediated immune regulation is scanty. In this study, we found that ATO improved antitumor immune response in vivo, which was associated with suppression of cell surface PD-L1 expression and EV PD-L1 levels in breast cancer cells.

In our previous study, sulfisoxazole and macitentan, which target endothelin receptor A, inhibited the secretion of tumor-derived EV PD-L1, but not the expression of cellular PD-L1, by regulating EV secretion in breast cancer cells and mouse models [18,20,35]. Interestingly, in the present study, ATO decreased both EV secretion and cellular PD-L1 expression; consequently, it suppressed tumor-derived EV PD-L1. This could be a novel strategy for effective inhibition of EV PD-L1.

Statins reduce cholesterol biosynthesis by inhibiting the mevalonate pathway via HMGCR inhibition. HMGCR is highly expressed in breast cancer patients [4]. The overexpression of HMGCR and genes of the mevalonate pathway is correlated with poor prognosis of recurrence-free and overall survival in breast cancer [5]. In acute myeloid leukemia (AML) cells, HMGCR^+^ EV upregulates intracellular cholesterol levels and promotes AML cell proliferation [36]. Treatment with the HMGCR inhibitor simvastatin or small interfering RNA targeting HMGCR blocks chemotherapy-induced enhancement of EV secretion in AML cells [36]. However, the mechanistic interplay between the mevalonate pathway and EV secretion remains poorly understood. In this study, we found ATO decreased EV secretion by regulating Rab. Moreover, we observed that Rab27a prenylation was inhibited by ATO treatment, and its electrophoretic movement was slower than that of the normal protein. Protein prenylation has been intensively studied in relation to cancer. Prenylation creates a lipidated hydrophobic domain and thereby affects membrane attachment or protein–protein interactions, which are essential for the biological function of proteins. Prenylation is observed for several members of the Ras and Rho families of small guanosine triphosphatases (GTPases). Rab prenylation is catalyzed by geranylgeranyl transferase (GGTase) II, which is also referred to as Rab GGTase. Rab GGTase transfers two geranylgeranyl groups to the C-terminal cysteine residues of Rab proteins [37]. Elevated expression of Rab27 GTPases is associated with poor prognosis and cancer metastasis. Moreover, these GTPases promote tumor growth and metastasis by enhancing exosome secretion, which alters intracellular microRNA levels, expression of signaling molecules, and the tumor microenvironment [38,39]. Statin-mediated inhibition of HMGCR disturbs posttranslational protein prenylation. Membrane targeting of Rab GTPases is influenced by the prenylation motif [40]. Our results show that the inhibition of prenylation by ATO leads to poor functioning of Rab27a and suppresses EV release by reducing vesicular membrane transport. Further studies on mapping prenylation sites of Rab proteins would be required.

We observed that decreased EV secretion, cellular PD-L1 expression, and cytotoxic effects of T cells induced by ATO were reversed by MV treatment. However, further studies are required to corroborate them.

We demonstrated that ATO enhanced antitumor immunity to induce cancer cell death when they were co-cultured with PBMCs. Orally administered ATO (up to 100 mg/kg) has been reported to show antitumor effects against several cancers in immunodeficient nude mice [41,42,43,44]. We also noticed concentration-dependent antitumor effects of ATO in immunodeficient mice, but not in immunocompetent mice, when ATO was administered (10 mg/kg) at a relatively low concentration than that in the previous study. Therefore, the CD8^+^ T cell-mediated immune response is important for the antitumor effects of ATO. Moreover, ATO potentiated anti-PD-L1 antibody therapy in syngeneic tumor models, which correlated with enhanced granzyme B secretion from cytotoxic T cells. In a previous study, ATO modulated the function of activated T cells by downregulating multiple co-inhibitory receptors corresponding to increased interleukin-2 production in stimulated T cells [45]. In addition, by targeting Ras-activated mTOR signaling via ATO, co-inhibitory receptors (e.g., PD-1, CTLA-4, TIM-3, LAG-3, 2B4, TIGIT, and CD160) and their ligands (e.g., PDL-1 and galectin-9) were downregulated [45]. Therefore, ATO can regulate EV PD-L1-mediated immune evasion by downregulating co-inhibitory receptors and enhancing effector T cells in the immune cells. Here, we present a novel mechanism whereby ATO improves EV PD-L1-mediated immunosuppression; in addition, our results provide evidence that ATO-induced antitumor immune responses can be regulated through the inhibition of co-inhibitory receptors owing to T-cell activity and can be correlated with inhibition of tumor-derived EV PD-L1.

In summary, ATO enhances the antitumor immune response and suppresses EV PD-L1 expression by reducing the expression of cell surface PD-L1 and EV secretion; this thereby inhibits the immune evasion of T cells. We showed that the mevalonate pathway is an important molecular link between ATO and EV PD-L1-dependent immune escape processes, indicating a potential therapeutic target for breast cancer treatment. Therefore, co-treatment of breast cancer patients with ATO and anti-PD-L1 antibodies may improve ICB responses. Importantly, this strategy can be used in future clinical trials for patients with breast cancer.

## Data Availability

All other relevant data of this study are available from the corresponding authors upon reasonable request.

## References

[1] Ribas A., Wolchok J.D. (2018). Cancer immunotherapy using checkpoint blockade. Science.

[2] Chen G., Huang A.C., Zhang W., Zhang G., Wu M., Xu W., Yu Z., Yang J., Wang B., Sun H. (2018). Exosomal PD-L1 contributes to immunosuppression and is associated with anti-PD-1 response. Nature.

[3] Sun J.-Y., Zhang D., Wu S., Xu M., Zhou X., Lu X.-J., Ji J. (2020). Resistance to PD-1/PD-L1 blockade cancer immunotherapy: Mechanisms, predictive factors, and future perspectives. Biomark. Res..

[4] Bjarnadottir O., Feldt M., Inasu M., Bendahl P.-O., Elebro K., Kimbung S., Borgquist S. (2020). Statin use, HMGCR expression, and breast cancer survival–The Malmö Diet and Cancer Study. Sci. Rep..

[5] Kimbung S., Lettiero B., Feldt M., Bosch A., Borgquist S. (2016). High expression of cholesterol biosynthesis genes is associated with resistance to statin treatment and inferior survival in breast cancer. Oncotarget.

[6] Baek A.E., Krawczynska N., Das Gupta A., Dvoretskiy S.V., You S., Park J., Deng Y.-H., Sorrells J.E., Smith B.P., Ma L. (2021). The cholesterol metabolite 27HC increases secretion of extracellular vesicles which promote breast cancer progression. Endocrinology.

[7] Wang H.-Y., Yu P., Chen X.-S., Wei H., Cao S.-J., Zhang M., Zhang Y., Tao Y.-G., Cao D.-S., Qiu F. (2022). Identification of HMGCR as the anticancer target of physapubenolide against melanoma cells by in silico target prediction. Acta Pharmacol. Sin..

[8] Jiang P., Mukthavaram R., Chao Y., Nomura N., Bharati I., Fogal V., Pastorino S., Teng D., Cong X., Pingle S. (2014). In vitro and in vivo anticancer effects of mevalonate pathway modulation on human cancer cells. Br. J. Cancer.

[9] Iannelli F., Lombardi R., Milone M.R., Pucci B., De Rienzo S., Budillon A., Bruzzese F. (2018). Targeting mevalonate pathway in cancer treatment: Repurposing of statins. Recent Pat. Anti-Cancer Drug Discov..

[10] Emberson J.R., Kearney P.M., Blackwell L., Newman C., Reith C., Bhala N., Holland L., Peto R., Keech A., Collins R. (2012). Lack of effect of lowering LDL cholesterol on cancer: Meta-analysis of individual data from 175,000 people in 27 randomised trials of statin therapy. PLoS ONE.

[11] Zheng X., Cui X.-X., Gao Z., Zhao Y., Lin Y., Shih W.J., Huang M.-T., Liu Y., Rabson A., Reddy B. (2010). Atorvastatin and celecoxib in combination inhibits the progression of androgen-dependent LNCaP xenograft prostate tumors to androgen independence. Cancer Prev. Res..

[12] Ghosh-Choudhury N., Mandal C.C., Ghosh-Choudhury N., Choudhury G.G. (2010). Simvastatin induces derepression of PTEN expression via NFκB to inhibit breast cancer cell growth. Cell. Signal..

[13] Cantini L., Pecci F., Hurkmans D., Copparoni C., Aerts S., Belderbos R.A., Cornelissen R., Dumoulin D.P., Fiordoliva I., Rinaldi S. (2020). Statin treatment improves response to anti-PD1 agents in patients with malignant pleural mesothelioma and non-small cell lung cancer. J. Clin. Oncol..

[14] Takada K., Shimokawa M., Takamori S., Shimamatsu S., Hirai F., Tagawa T., Okamoto T., Hamatake M., Tsuchiya-Kawano Y., Otsubo K. (2022). A propensity score-matched analysis of the impact of statin therapy on the outcomes of patients with non-small-cell lung cancer receiving anti-PD-1 monotherapy: A multicenter retrospective study. BMC Cancer.

[15] Lim W.-J., Lee M., Oh Y., Fang X.-Q., Lee S., Lim C.-H., Park J., Lim J.-H. (2021). Statins Decrease Programmed Death-Ligand 1 (PD-L1) by inhibiting AKT and β-Catenin signaling. Cells.

[16] Kulshreshtha A., Singh S., Ahmad M., Khanna K., Ahmad T., Agrawal A., Ghosh B. (2019). Simvastatin mediates inhibition of exosome synthesis, localization and secretion via multicomponent interventions. Sci. Rep..

[17] Hyder T., Marti J.L.G., Nasrazadani A., Brufsky A.M. (2021). Statins and endocrine resistance in breast cancer. Cancer Drug Resist..

[18] Im E.-J., Lee C.-H., Moon P.-G., Rangaswamy G.G., Lee B., Lee J.M., Lee J.-C., Jee J.-G., Bae J.-S., Kwon T.-K. (2019). Sulfisoxazole inhibits the secretion of small extracellular vesicles by targeting the endothelin receptor A. Nat. Commun..

[19] Théry C., Amigorena S., Raposo G., Clayton A. (2006). Isolation and characterization of exosomes from cell culture supernatants and biological fluids. Curr. Protoc. Cell Biol..

[20] Lee C.-H., Bae J.-H., Choe E.-J., Park J.-M., Park S.-S., Cho H.J., Song B.-J., Baek M.-C. (2022). Macitentan improves antitumor immune responses by inhibiting the secretion of tumor-derived extracellular vesicle PD-L1. Theranostics.

[21] Théry C. (2014). Biogenesis, secretion, and intercellular interactions of exosomes and other extracellular vesicles. Annu. Rev. Cell Dev. Biol..

[22] Detter J.C., Zhang Q., Mules E.H., Novak E.K., Mishra V.S., Li W., McMurtrie E.B., Tchernev V.T., Wallace M.R., Seabra M.C. (2000). Rab geranylgeranyl transferase α mutation in the gunmetal mouse reduces Rab prenylation and platelet synthesis. Proc. Natl. Acad. Sci. USA.

[23] Peng P., Wei W., Long C., Li J. (2017). Atorvastatin augments temozolomide’s efficacy in glioblastoma via prenylation-dependent inhibition of Ras signaling. Biochem. Biophys. Res. Commun..

[24] Wu Y., Chen W., Xu Z.P., Gu W. (2019). PD-L1 distribution and perspective for cancer immunotherapy—Blockade, knockdown, or inhibition. Front. Immunol..

[25] Roberts P.J., Der C.J. (2007). Targeting the Raf-MEK-ERK mitogen-activated protein kinase cascade for the treatment of cancer. Oncogene.

[26] Hu T., Shen H., Huang H., Yang Z., Zhou Y., Zhao G. (2020). Cholesterol-lowering drug pitavastatin targets lung cancer and angiogenesis via suppressing prenylation-dependent Ras/Raf/MEK and PI3K/Akt/mTOR signaling. Anti-Cancer Drugs.

[27] Beckwitt C.H., Brufsky A., Oltvai Z.N., Wells A. (2018). Statin drugs to reduce breast cancer recurrence and mortality. Breast Cancer Res..

[28] Mo H., Jeter R., Bachmann A., Yount S.T., Shen C.-L., Yeganehjoo H. (2019). The potential of isoprenoids in adjuvant cancer therapy to reduce adverse effects of statins. Front. Pharmacol..

[29] Kim H., Seol Y.M., Choi Y.J., Shin H.-J., Chung J.S., Shin N., Kim A., Kim J.Y., Kim K.Y., Bae Y. (2019). HMG CoA reductase expression as a prognostic factor in Korean patients with breast cancer: A retrospective study. Medicine.

[30] Gruenbacher G., Thurnher M. (2015). Mevalonate metabolism in cancer. Cancer Lett..

[31] Kantor E.D., Lipworth L., Fowke J.H., Giovannucci E.L., Mucci L.A., Signorello L.B. (2015). Statin use and risk of prostate cancer: Results from the Southern Community Cohort Study. Prostate.

[32] Freedland S., Hamilton R., Gerber L., Banez L., Moreira D., Andriole G., Rittmaster R. (2013). Statin use and risk of prostate cancer and high-grade prostate cancer: Results from the REDUCE study. Prostate Cancer Prostatic Dis..

[33] Mullen P.J., Yu R., Longo J., Archer M.C., Penn L.Z. (2016). The interplay between cell signalling and the mevalonate pathway in cancer. Nat. Rev. Cancer.

[34] Maisano D., Mimmi S., Russo R., Fioravanti A., Fiume G., Vecchio E., Nisticò N., Quinto I., Iaccino E. (2020). Uncovering the exosomes diversity: A window of opportunity for tumor progression monitoring. Pharmaceuticals.

[35] Nishida-Aoki N., Tominaga N., Takeshita F., Sonoda H., Yoshioka Y., Ochiya T. (2017). Disruption of circulating extracellular vesicles as a novel therapeutic strategy against cancer metastasis. Mol. Ther..

[36] Hong C.-S., Jeong E., Boyiadzis M., Whiteside T.L. (2020). Increased small extracellular vesicle secretion after chemotherapy via upregulation of cholesterol metabolism in acute myeloid leukaemia. J. Extracell. Vesicles.

[37] Thurnher M., Nussbaumer O., Gruenbacher G. (2012). Novel aspects of mevalonate pathway inhibitors as antitumor agents. Clin. Cancer Res..

[38] Bobrie A., Krumeich S., Reyal F., Recchi C., Moita L.F., Seabra M.C., Ostrowski M., Théry C. (2012). Rab27a supports exosome-dependent and-independent mechanisms that modify the tumor microenvironment and can promote tumor progression. Cancer Res..

[39] Li Z., Fang R., Fang J., He S., Liu T. (2018). Functional implications of Rab27 GTPases in cancer. Cell Commun. Signal..

[40] Gomes A.Q., Ali B.R., Ramalho J.S., Godfrey R.F., Barral D.C., Hume A.N., Seabra M.C. (2003). Membrane targeting of Rab GTPases is influenced by the prenylation motif. Mol. Biol. Cell.

[41] Iotefa T., Guillaume S., Liza F., Véronique M., Philippe R. (2016). Atorvastatin-Induced Inhibition of Human Melanoma. Immunother. Open Access.

[42] Dai X.-Y., Zhuang L.-H., Wang D.-D., Zhou T.-Y., Chang L.-L., Gai R.-H., Zhu D.-F., Yang B., Zhu H., He Q.-J. (2016). Nuclear translocation and activation of YAP by hypoxia contributes to the chemoresistance of SN38 in hepatocellular carcinoma cells. Oncotarget.

[43] Kubatka P., Bojková B., Kassayová M., Orendáš P., Kajo K., Výbohová D., Kružliak P., Adamicová K., Péč M., Stollárová N. (2014). Combination of Pitavastatin and melatonin shows partial antineoplastic effects in a rat breast carcinoma model. Acta Histochem..

[44] Kitagawa K., Moriya K., Kaji K., Saikawa S., Sato S., Nishimura N., Namisaki T., Akahane T., Mitoro A., Yoshiji H. (2020). Atorvastatin augments gemcitabine-mediated anti-cancer effects by inhibiting Yes-associated protein in human cholangiocarcinoma cells. Int. J. Mol. Sci..

[45] Okoye I., Namdar A., Xu L., Crux N., Elahi S. (2017). Atorvastatin downregulates co-inhibitory receptor expression by targeting Ras-activated mTOR signalling. Oncotarget.

